# Bad bugs mean business: gastric cancer is an intended consequence of *Helicobacter pylori* infection

**DOI:** 10.1128/iai.00032-26

**Published:** 2026-07-13

**Authors:** Valerie P. O'Brien

**Affiliations:** 1 Borch Department of Medicinal Chemistry and Molecular Pharmacology, Purdue University College of Pharmacy 15483 , West Lafayette, Indiana, USA; University of Pittsburgh, Pittsburgh, Pennsylvania, USA

**Keywords:** *Helicobacter pylori*, gastric cancer, metaplasia, pathogen-associated malignancy

## Abstract

The bacterium *Helicobacter pylori* (*Hp*) infects half the world and causes stomach inflammation. Most infections are asymptomatic, but in 1%–2% of infected individuals, inflammation can lead to a cascade of tissue changes that culminates in the development of metaplasia (pre-cancer) and finally gastric cancer, a highly fatal disease. It has been posited that *Hp*’s role in the process is merely to initiate inflammation, and once the later stages of disease develop, *Hp* becomes dispensable. However, epidemiological and molecular studies support a different paradigm: that in at least some individuals, *Hp* actively drives metaplasia and carcinogenesis. Molecular profiling shows that *Hp* is prevalent during gastric cancer. In individuals with metaplasia, successful antibiotic eradication of *Hp* prevents the development of gastric cancer; antibiotic eradication after cancer appears can even significantly reduce the likelihood of tumor recurrence. In rodent models of gastric cancer, *Hp* infection accelerates carcinogenesis, and in a metaplasia model, *Hp* infection alters the metaplastic lineages present and causes increased dysplasia (abnormal cells). Taken together, these data point to an active role for *Hp* infection in driving gastric cancer development, at least in some individuals. Therefore, studies investigating host-pathogen interactions in the context of *Hp*-driven carcinogenesis should encompass the entire spectrum of disease development, not just the establishment of gastric infection and initiation of inflammation. Understanding the complete picture of *Hp*-driven disease will enable us to tackle this highly fatal cancer.

## *HELICOBACTER PYLORI*: A BACTERIAL CARCINOGEN

There were almost one million new gastric (stomach) cancer cases and more than 660,000 new deaths from the disease worldwide in 2022 (1), with tens of thousands occurring in the United States (2). Globally, about 80% of gastric cancer cases are attributable to infection with *Helicobacter pylori* (*Hp*) (3), a bacterium that colonizes the stomach of half of the world’s population (4). *Hp* infection typically occurs during childhood, is usually lifelong, and always causes inflammation of the stomach lining (gastritis). Gastritis is usually asymptomatic and does not lead to serious sequelae. However, *Hp* infection leads to peptic or duodenal ulcers in 10%–20% of individuals, gastric cancer in 1%–2% of individuals, and mucosal-associated lymphoid tissue (MALT) lymphoma in <1% of individuals (5, 6).

**Fig 1:**
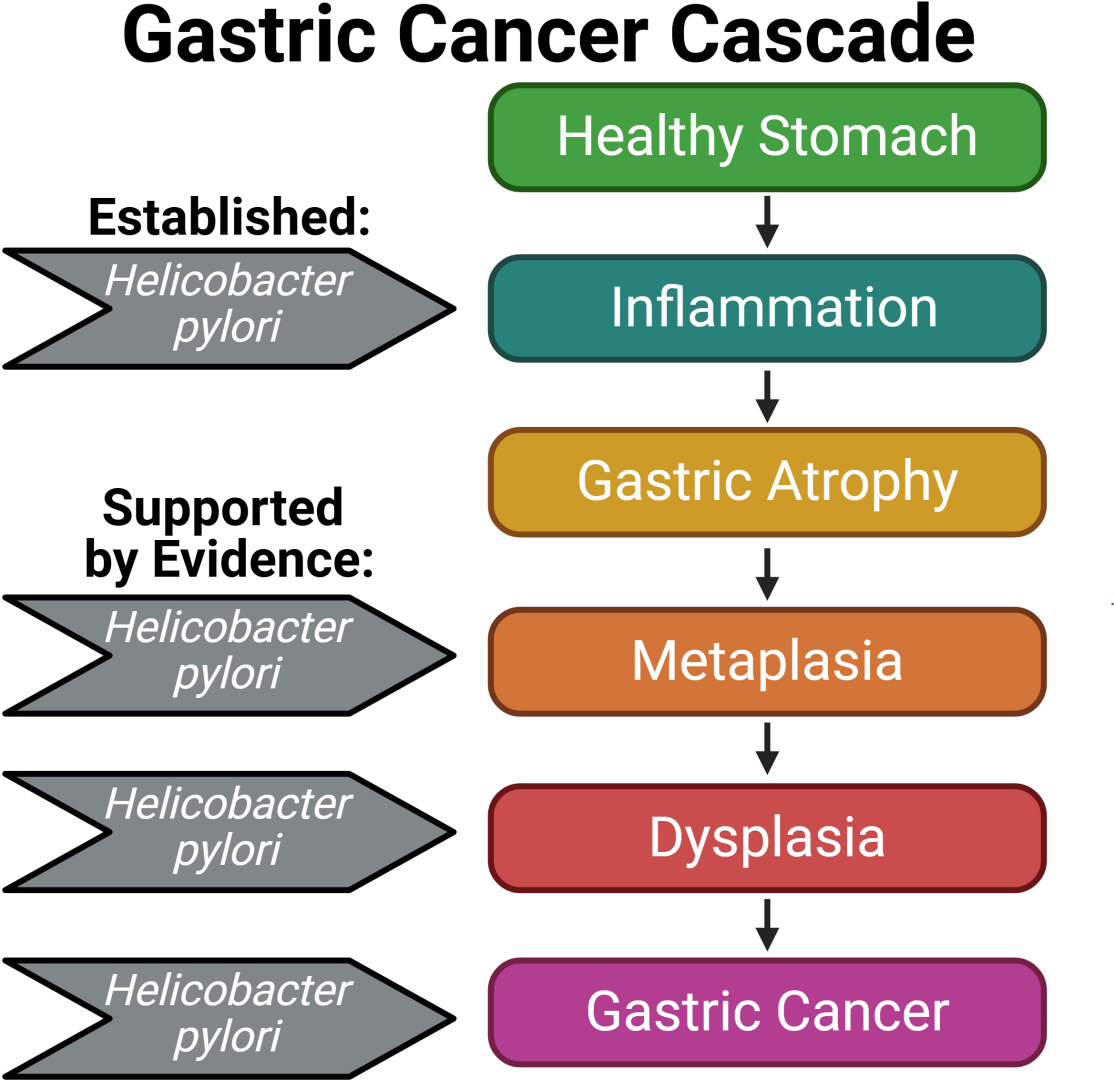
*Helicobacter pylori* (*Hp*) infection promotes gastric cancer development. The so-called Correa cascade of gastric cancer development is shown. *Hp* is well established as the trigger of gastric inflammation (gastritis). However, evidence from epidemiological studies of disease progression in antibiotic-treated human subjects, molecular analyses of *Hp* stomach colonization during gastric cancer, and mechanistic studies in rodent models demonstrate that *Hp* can actively promote the later stages of the cascade: metaplasia, dysplasia, and cancer development and recurrence. Note that while this figure depicts atrophy, metaplasia, and dysplasia as a stepwise process, this is a commonly employed oversimplification of the disease cascade (for example, as seen in reference 19). Although these tissue changes are assessed and graded separately by pathologists, they may co-occur within the same patient (8, 20). Atrophy, metaplasia, and dysplasia are all considered precursor lesions to gastric cancer (21). Created in BioRender. O'Brien, V. (2026) https://BioRender.com/1e5b47l.

Pathologist Pelayo Correa’s work helped define the histological changes that precede gastric cancer development (7)—the so-called “Correa cascade” (Fig. 1). Inflammation in the stomach initially manifests as superficial gastritis, which in some individuals can progress to gastric atrophy, the loss of appropriate glands in the gastric epithelium (8). Two phenotypes of atrophy can occur: non-metaplastic atrophy and metaplastic atrophy. Non-metaplastic atrophy is the shrinking or vanishing of gastric glands coupled with the expansion of the glandular lamina propria. Metaplastic atrophy is the replacement of native glands by glands with new cell types, a phenomenon termed metaplasia (9). Metaplastic atrophy is termed “gastric intestinal metaplasia” when native glands are replaced by intestinalized glands that contain cells of the small or large intestine, such as goblet cells. Alternatively, the native glands of the oxyntic mucosa (the gastric acid-producing region of the stomach) may be replaced by mucus-secreting glands like those of the distal stomach, which is often called “pseudopyloric metaplasia” (10). Both metaplastic and non-metaplastic atrophy are considered precursor lesions to gastric cancer, as is dysplasia, the presence of abnormal cells. Among these precursor lesions (together termed “preneoplasia”), the diagnosis that is most strongly associated with gastric cancer development is dysplasia. For example, in a nationwide cohort study in the Netherlands, within 10 years of an initial diagnosis of a precursor lesion, gastric cancer was diagnosed in 0.8% of subjects with gastric atrophy; 1.8% of subjects with intestinal metaplasia; 3.9% of subjects with mild-to-moderate dysplasia; and 32.7% of subjects with severe dysplasia (11). Correa and colleagues established this trajectory based on many years of analysis of human specimens (12), and while they clearly noted the strong link between inflammation and preneoplastic progression in the stomach, with inflammation present at every stage of the pre-cancer cascade (12), the cause of the inflammation remained elusive. Barry Marshall and J. Robin Warren’s Nobel Prize-winning discovery of *Hp* as a major cause of gastritis and peptic ulcer disease (13) sparked a revolution in gastric cancer research. *Hp* was soon linked to intestinal metaplasia (14) and gastric cancer (15–18), and in 1994, the International Agency for Research on Cancer working group of the World Health Organization categorized *Hp* as a Group I (known) carcinogen (17).

*Hp* is a highly genetically diverse gram-negative bacterium. It possesses many virulence factors that have been previously reviewed (6, 22, 23). *Hp* triggers a strong T cell response that controls but does not eradicate the infection (24–26); T cells can also drive gastric immunopathology (27, 28). Other factors contributing to gastric cancer risk include alcohol and tobacco use, male sex, and host genetics (6, 29). *Hp* is acid-tolerant, but *Hp*-driven gastric atrophy causes the loss of acid-producing parietal cells, which can increase the pH of the stomach, reducing the acid barrier that prevents most other bacteria from colonizing this niche. Therefore, *Hp* may be out-competed and lost from the stomach as the disease progresses. This observation has led to confusion about *Hp*’s role in the later stages of the Correa cascade, with some proposing that *Hp*’s primary role is merely to initiate inflammation (30–34). Although *Hp* infection causes gastric cancer, this is clearly a rare consequence of infection, occurring in only about 1%–2% of infected individuals (5, 6); most infections are asymptomatic. Furthermore, gastric cancer is highly fatal, and because *Hp* depends on its host for survival and transmission, death of the host would be disadvantageous to *Hp*. Together, these observations could paint the picture of gastric cancer as an unintended consequence of *Hp* infection. In this review, I will highlight evidence from epidemiological studies, molecular profiling of gastric samples, and mouse carcinogenesis models that, taken together, paint a different picture: *Hp* actively drives disease progression, at least in some individuals.

## *HP* IS OFTEN PRESENT DURING GASTRIC CANCER

Given that *Hp* may be lost in the later stages of disease as the changing gastric environment favors outgrowth of other bacteria (33), an assessment of the prevalence of *Hp* in individuals with gastric cancer is warranted. Below, I will highlight several studies that have used various molecular methods to address this question.

Polymerase chain reaction (PCR) was used to amplify the *Hp* urease gene in a collection of 109 gastric carcinoma specimens collected in China and yielded a positive result in 53.1% of frozen tissues vs 32.5% of paraffin-embedded tissues (35), suggesting that tissue preservation methods have a strong impact on downstream *Hp* detection. The same samples were used for *Hp* detection by Warthin-Starry staining, followed by imaging. Intriguingly, the authors reported that *Hp* cells could be seen both in the tumor tissue as well as in adjacent non-neoplastic tissue, though the bacteria were significantly more likely to be found in non-neoplastic tissue than in tumors. Significantly more samples tested positive by staining (53.2%) than by PCR (38.5%). The majority of samples had concordant staining and PCR results, but 18.3% were negative by PCR and positive by staining, whereas 3.7% were positive by PCR and negative by staining. Interestingly, when *Hp* status was assessed according to the cancer stage, 12 of 16 early-stage carcinomas (75%) were *Hp*-positive by staining, whereas only 46 of 93 advanced carcinomas (49.5%) were *Hp*-positive; the same trend was seen for cases that were positive by PCR (35). Although there were far fewer early-stage samples in this study, this observation suggests that *Hp* infection may be lost as cancer progresses from early to later stages.

In another study of Chinese subjects (36), 166 individuals with gastric cancer or undergoing endoscopy for other indications were screened for active *Hp* infection using the urea breath test, a method that detects *Hp* based on its ability to hydrolyze radiolabeled urea (37). Among subjects with gastric cancer, 25 of 58 (43%) had active *Hp* infection, compared to 25 of 108 subjects without gastric cancer (23%) (36). The *Hp*-positive individuals provided gastric brushings and stool samples for detection of *Hp* by droplet digital PCR, a more sensitive and specific method than classical PCR, and biopsy specimens for *Hp* detection by Wright-Giemsa staining. In 89% of subjects, all three methods successfully detected *Hp. Hp* loads in the stomach were not significantly different between individuals with vs without gastric cancer or with vs without intestinal metaplasia. Interestingly, individuals with gastric cancer had significantly higher *Hp* loads in the stool than those without cancer. Given that the fecal-oral route is one proposed route of *Hp* transmission (38), a higher *Hp* stool load during cancer could potentially allow for increased transmission to new hosts, though this intriguing concept is speculative and would require experimental and epidemiological validation.

The Asian Cancer Research Group profiled 300 gastric cancers to investigate potential tumor molecular subtypes (39). Based on gene expression signatures, tumors were categorized as follows: microsatellite stable with TP53 functional loss (35.7%); microsatellite stable with intact TP53 activity (26.3%); microsatellite stable with a signature of epithelial-to-mesenchymal transition (15.3%); and microsatellite instability (22.7%). A subset of 127 cases were assessed for *Hp* infection by histology staining of gastric biopsies; of these, 42.5% (*n* = 55) were positive and were evenly distributed among the four tumor molecular subtypes. In a similar study, The Cancer Genome Atlas (TCGA) profiled 295 gastric tumors and proposed four molecular subtypes (39). The investigators “detected only sporadic evidence of [*Hp*]” by analyzing tumor whole-genome and whole-exome sequencing reads with PathSeq, a computational pipeline that can identify microbial signatures in human sequencing data by discarding reads that map to the human genome and searching for the remaining reads in microbial sequence databases (40). We now appreciate the limitations of PathSeq, including the observation that microbial sequence databases are frequently contaminated with human DNA, leading to false positives (41). A more stringent analysis of microbial signatures in TCGA data using KrakenUniq found that *Hp* was the 17th most common microbe in gastric cancer samples (42). More broadly, the TCGA study illustrates the limitations in detecting *Hp* through non-specific whole-genome and whole-exome sequencing methods.

Together, these studies show that *Hp* typically can be detected in about 40%–50% of gastric cancer patients. Multiple modalities should be used to detect *Hp* as a given sample may be positive by one method and negative by another. Additional studies are needed to clarify whether *Hp* decreases in abundance or is lost entirely as the cancer progresses. Notably, other than the TCGA study, the investigations described above used samples from Chinese and Korean populations, which have high *Hp* infection prevalence and, consequently, high gastric cancer incidence (43). We recently profiled fixed tissue samples from 47 subjects with gastric cancer treated at medical centers in the United States Pacific Northwest. Using droplet digital PCR, we detected *Hp* in only 11 of 47 subjects (23%). It is likely that *Hp* prevalence during gastric cancer is heterogeneous in different populations and is impacted by various factors known to impact the risk of *Hp* infection and/or gastric cancer development, such as bacterial virulence factors, host polymorphisms in immune response genes, and diet (29). Nonetheless, it is clear that many individuals with gastric cancer still harbor active *Hp* infection. But by the time tumors develop in these individuals, is *Hp* still an active participant in the disease process or merely a bystander? Epidemiological studies evaluating the impact of antibiotics on gastric cancer outcomes shed light on this question.

## ANTIBIOTICS PREVENT GASTRIC CANCER

Meta-analyses have clearly established that *Hp* eradication significantly reduces gastric cancer risk (44). Below, I will highlight a few studies of particular importance.

### The Veterans Health Administration (VHA) study

A large retrospective cohort study evaluated gastric cancer incidence in 371,813 United States military veterans seen at the VHA who received a diagnosis of *Hp* infection during a 25-year period (45). The overall incidence of gastric cancer within *Hp*-positive individuals was 0.65% after 20 years, with a higher incidence among Asian, Black/African American, and Hispanic individuals. Intriguingly, the incidence of gastric cancer in *Hp*-positive individuals in this study was about fivefold lower than in Japan, a country with high *Hp* infection prevalence and gastric cancer incidence; the high gastric cancer incidence in Japan can be partially attributed to the high prevalence of highly virulent *Hp* strains in most of the country, compared to less virulent, “Western-type” *Hp* strains (46). In the military veterans study, many individuals were apparently diagnosed based on serum antibody testing, even though this has been explicitly recommended against in the United States because the serum antibody test does not strictly reflect active infection; a serum antibody response can last well after the infection is cleared (47). *Hp* serology tests can also give false-negative results (48). Furthermore, 69.5% of subjects received *Hp* eradication therapy without any record of *Hp* diagnostic testing or serum antibody testing. While it is possible that some of these patients were tested outside the VHA system, it is likely that at least some individuals were prescribed eradication therapy empirically, perhaps based on gastric symptoms, without having active *Hp* infection. If so, this could help explain the comparatively low incidence of gastric cancer in the overall population, if many of these individuals did not actually have active *Hp* infection.

Critically, only 74.8% of subjects with a positive diagnostic test were even prescribed an eradication regimen, even though American College of Gastroenterology guidelines make it clear that all infected individuals should be offered eradication therapy (49, 50). Among these subjects, only 20.8% underwent retesting after treatment, even though, again, retesting is strongly recommended by clinical guidelines (49, 50). Of this small subset of the overall cohort, only 90.9% had successful eradication of *Hp*, underscoring the high intrinsic antimicrobial resistance among *Hp* isolates that makes eradication efforts difficult. Notably, among these individuals with confirmed active *Hp* infection based on a positive diagnostic test (i.e., stool antigen, urea breath test, or endoscopy, rather than a serum antibody test), those with confirmed eradication of the infection had a significant reduction in gastric cancer incidence compared to those without eradication (subhazard ratio: 0.24; 95% confidence interval: 0.15 to 0.41; *P* < 0.001). Thus, this landmark study confirmed that successful eradication of active *Hp* infection significantly reduces the likelihood of subsequent gastric cancer development, while also demonstrating several challenges associated with *Hp* detection and treatment.

### The Colombian chemoprevention trial

This study was a prospective cohort trial designed to test the hypothesis that curing *Hp* infection and supplementing dietary antioxidants could protect against gastric cancer development (51). Subjects were recruited from a region in Colombia where *Hp* infection and gastric cancer are common. All enrolled subjects had a diagnosis of gastric atrophy with or without intestinal metaplasia and dysplasia in endoscopic biopsy samples, and 97% were *Hp*-positive. Half of the subjects received *Hp* eradication therapy for 2 weeks. Subjects were randomly assigned to receive daily dietary supplements of ascorbic acid and/or β-carotene or control supplements for 6 years, with compliance highly encouraged and monitored by social workers. Ultimately, 852 subjects received their intended intervention, with 631 completing the initial 6-year trial. Intriguingly, in subjects with gastric atrophy without intestinal metaplasia at baseline (i.e., those with less severe disease), any combination of eradication therapy and/or antioxidants caused a statistically significant regression of the disease at the 6-year follow-up, compared to placebo-treated subjects who did not receive *Hp* eradication therapy. In subjects with multifocal gastric atrophy and intestinal metaplasia (i.e., those with more severe disease), only *Hp* eradication treatment caused a statistically significant decrease in disease progression and increase in regression.

What is remarkable about the Colombian Chemoprevention Trial is the very long follow-up period: additional gastric biopsy specimens were collected at 12, 16, and 20 years after enrollment, with 356 individuals completing the 20-year follow-up. After 6 years, *Hp* eradication therapy was offered to the initially untreated subjects, and at subsequent follow-up visits, tissue histology was assessed based on *Hp* status. A histopathology scoring system was used to quantify changes to the tissue. By the 12-year follow-up, there was no longer any impact of antioxidant treatment; only subjects initially assigned to receive *Hp* eradication therapy showed a significant change in histopathology, with a decrease of 0.28 units from the baseline score (52). In the first 12 years of the study, the rate of spontaneous *Hp* clearance was 2.9% per year, and the rate of *Hp* reinfection was 5.4% per year. Evaluation of histopathology scores based on *Hp* status was striking. Scores were similar at the 3-year follow-up, but by 6 years, scores began to decrease in individuals who were *Hp*-negative compared to those who were *Hp*-positive. In the *Hp*-negative individuals, scores further decreased at 12 years (52), demonstrating that cumulative time without *Hp* infection promotes regression of disease. Multivariate modeling of disease progression conducted at the 16-year follow-up estimated that continuous *Hp* infection increased the average histopathology score by 0.2 units per year (53). Intriguingly, the 20-year follow-up showed that the average histopathology score increased between 12 and 20 years regardless of *Hp* status, which might have been be due to accumulation of damage in the gastric mucosa due to dietary and environmental exposures. However, the significant gap between *Hp*-negative and *Hp*-positive individuals was steady (54). Thus, this long-term study convincingly shows that sustained *Hp* infection leads to disease progression, whereas successful, long-term *Hp* eradication prevents gastric disease progression.

### The Matsu Islands study of mass eradication

In gastric cancer, the “point of no return” is when oncogenic changes in the stomach can no longer be reversed and cancer is inevitable (55). A large study on the Matsu Islands in Taiwan initially found no reduction in intestinal metaplasia prevalence or severity 4 years after a mass chemoprevention effort, compared to the prevalence of intestinal metaplasia several years before the chemoprevention program was implemented. When intestinal metaplasia was assessed based on *Hp* status, a reduction was seen in individuals with successful eradication, but the difference was not statistically significant (56). However, when gastric disease was assessed on the Matsu Islands 14 years later, at the conclusion of the mass *Hp* eradication program, the prevalence of advanced-stage (moderate or marked) gastric atrophy had decreased from 7.1% to 0% and the prevalence of advanced-stage intestinal metaplasia had decreased from 11.8% to 1.8% (57). Thus, this long-term study once again demonstrates that *Hp* eradication prevents preneoplastic progression. At the 4-year follow-up, the authors declared that *Hp* eradication “does not reduce the incidence of intestinal metaplasia or decrease its histological severity, supporting the ‘point-of-no-return’ theory.” (56) This finding, of course, would prove incorrect over a longer follow-up period and thus underscores the need to consider study duration when evaluating the results of human trials. Notably, long-term follow-up revealed that the mass *Hp* eradication program resulted in a 53% reduction in gastric cancer incidence compared to historical control data and is predicted to achieve a 68% reduction in incidence and 39% reduction in mortality as a longer follow-up period is achieved (57).

### Antibiotics during gastric cancer

A South Korean clinical trial with 396 participants tested whether antibiotic eradication of *Hp* could prevent metachronous gastric cancer, a new primary gastric cancer that develops at least 1 year after the initial primary cancer (58). Subjects with early gastric cancer or high-grade adenoma plus active *Hp* infection were randomly assigned to receive either antibiotics after endoscopic resection of the cancer or placebo treatment after endoscopic resection and were followed-up for a median duration of almost 6 years. *Hp* infection status was assessed 3 months after treatment: only 80.4% of treated subjects were *Hp*-negative at this time, again emphasizing the high inherent antibiotic resistance of *Hp*. Interestingly, 5.4% of placebo-treated subjects tested negative at this time as well. Only 5.4% of subjects with eradicated *Hp* infection developed metachronous gastric cancer, compared to 14.0% of subjects with persistent *Hp* infection, a significant difference. The authors also evaluated tissue biopsies collected at baseline and 3 years later in a subset of 327 subjects with available specimens. In antibiotic-treated subjects, one region of the stomach, the lesser curvature of the corpus, showed a statistically significant improvement in the extent of atrophy (48.4% had improvement, compared to 15.0% of placebo-treated subjects) and intestinal metaplasia (36.6% showed improvement, compared to 18.3% of placebo-treated subjects). Other studies with different designs have drawn similar conclusions about metachronous gastric cancer. In an open-label randomized controlled trial with a 3-year follow-up period, metachronous gastric cancer occurred in 3.3% (9 of 272) of the subjects in the eradication group and 8.8% (24 of 272) of the control group, a significant difference (59). Meta-analyses of eight cohort studies again found that the overall incidence of metachronous gastric cancer was significantly reduced in individuals who received antibiotic therapy to eradicate *Hp* (44). However, in an open-label randomized controlled trial with a median follow-up period of 3 years, metachronous gastric cancer occurred in 2.3% (10 of 444) of subjects who received antibiotic therapy and 3.7% (17 of 457) of non-antibiotic-treated subjects; in this trial, the difference was not statistically significant, but the overall incidence of metachronous gastric cancer was low (60). Overall, these studies show that successful antibiotic eradication of active *Hp* infection during early-stage gastric cancer reduces metachronous gastric cancer and allows the stomach to heal.

### A point of no return?

These studies convincingly show that successful *Hp* eradication significantly prevents preneoplastic progression and even gastric cancer recurrence—but not in every individual. For example, the 20-year follow-up of the Colombian Chemoprevention Trial showed that 9% of the individuals with intestinal metaplasia at baseline progressed to dysplasia or gastric cancer during the follow-up period (54), and in the South Korean trial of *Hp* eradication to prevent metachronous cancer, 5.4% of subjects with successfully eradicated *Hp* infection still developed metachronous gastric cancer after resection of the initial cancer (58). These individuals represent the so-called “point of no return,” beyond which gastric cancer will develop regardless of *Hp* eradication (55). Therefore, there is still a critical clinical need to develop novel gastric cancer prevention and treatment strategies.

A landmark study performed epigenomic profiling of metaplasia and blood samples from subjects enrolled in a decade-long prospective trial (61). During the study period, most subjects had no change in disease status. However, some subjects progressed to gastric cancer; their initial biopsies had somatic copy number alterations and significantly shorter telomere lengths compared to biopsies from individuals with stable disease. Also, some subjects regressed to less severe metaplasia or even no metaplasia; their initial biopsies had a lower mutational burden and were less methylated than biopsies from individuals with stable disease. The authors hypothesized that methylation levels might influence the “point of no return” (61). Another study evaluated cancer-associated phenotypes in gastric biopsy samples collected before and after confirmed eradication of *Hp* infection and found that eradication reduced the activation of the DNA damage response but did not decrease the number of senescent cells (62). While specimens were evaluated at a relatively short time point after eradication (at least 2 months later, with an average of 434 days between endoscopies), sustained senescence after *Hp* eradication is an intriguing finding as senescence has been hypothesized to promote gastric atrophy (63) and various gastrointestinal cancers (64). Whether aberrant methylation (or other epigenomic alterations) and/or cellular senescence could drive the “point of no return” would be of great importance to assess with mechanistic molecular studies. However, we must remember that this phenomenon impacts only a minority of the population. The key takeaway is that on a population level, antibiotic eradication of *Hp* during metaplasia prevents stomach cancer development, and eradication during cancer treatment prevents stomach cancer from recurring. Thus, *Hp* is clearly important for both preneoplastic progression and tumorigenesis.

## *HELICOBACTER* INFECTION ACCELERATES CARCINOGENESIS IN MOUSE MODELS

The studies described above clearly demonstrate that in at least some individuals, *Hp* actively colonizes the stomach even after cancer develops, and that successful antibiotic eradication of *Hp* significantly reduces (but does not eliminate) all aspects of disease progression, from gastric atrophy to metaplasia and cancer to tumor recurrence after resection. Together, these key points paint a different picture of gastric carcinogenesis: that in at least some individuals, *Hp* plays an active role in driving all stages of disease. Evidence from rodent models supports this hypothesis. In wild-type mice, *Hp* can cause metaplasia in a manner dependent on the bacterial strain and mouse age at the time of infection (65), but it does not cause cancer for unknown reasons (66). In contrast, *Hp* infection of Mongolian gerbils does cause gastric cancer (67). Therefore, mouse models commonly harness oncogene expression and/or exposure to chemical carcinogens to induce more severe metaplasia phenotypes and gastric cancer (68). Below, I highlight studies that incorporated *Helicobacter* infection into rodent models of gastric carcinogenesis. These studies demonstrate that *Helicobacter* alters and/or exacerbates the course of disease. Note that some studies used *Helicobacter felis* (*Hf*) rather than *Hp*; *Hf* induces more severe inflammation and metaplasia in mice (69) and therefore can be useful in carcinogenesis models, although it is arguably less clinically relevant than the human pathogen *Hp*.

### INS-GAS gastric cancer model

Gastrin is a hormone that stimulates gastric acid secretion. INS-GAS mice overexpress human gastrin from a rat insulin promoter, causing hypergastrinemia (70) that initially causes increased acid secretion but ultimately triggers loss of acid-producing parietal cells and metaplasia, which progresses to gastric cancer by 20 months of age (71). Infection of INS-GAS mice with *Helicobacter* species has been a critical tool for uncovering mechanisms of gastric carcinogenesis. *Hf* infection causes significant gastric inflammation (72) and accelerates gastric cancer development, with 85% of *Hf*-infected INS-GAS mice developing gastric carcinoma within 7 months (71). *Hp* infection also accelerates disease progression in this model. In male mice, *Hp* infection causes metaplasia and severe dysplasia within 7 months, with ~67% of the mice developing gastric cancer (73). In female mice, disease is less severe and does not progress to cancer within this time frame due to a protective effect from female sex hormones (74). Gastric cancer is about twice as common in men than women (75), bolstering the clinical significance of this model. In accordance with the protective effect seen with *Hp* eradication in humans, *Hp* eradication in INS-GAS mice significantly attenuates disease progression; while the effect was strongest for mice treated at early stages of infection, even mice treated at later time points had less severe dysplasia compared to untreated, *Hp*-infected mice (76). However, one study unexpectedly found that antibiotic treatment significantly reduced disease severity even in mock-infected INS-GAS mice, suggesting that commensal microbes could play a role in disease pathogenesis in this model (77). Several studies have evaluated this hypothesis using germ-free INS-GAS mice. Strikingly, gastric cancer did not develop in germ-free INS-GAS mice euthanized at 13 months of age, demonstrating that commensal microbes do indeed contribute to disease progression in this model. In the same study, gastric cancer incidence was also reduced in *Hp*-monoassociated (gnotobiotic) INS-GAS mice compared to *Hp*-infected, conventionally housed INS-GAS mice: only 44% of male and 8% of female gnotobiotic mice developed cancer by 11 months post-infection (78). Another study showed that colonization of germ-free INS-GAS mice with just three species of altered Schaedler’s flora was sufficient to promote gastric pathology and augment *Hp*-associated carcinogenesis (79). More recently, different bacteria have shown different effects on *Hp*-associated disease progression in gnotobiotic INS-GAS mice. *Streptococcus salivarius* augmented *Hp*-associated disease, whereas *Staphylococcus epidermidis* did not impact *Hp*-associated disease (80), and in female mice, infection with thiopeptide-producing *Cutibacterium acnes* prior to *Hp* significantly increased dysplasia compared to *Hp* infection alone (81). Thus, commensal microbes clearly have a significant impact on carcinogenesis in *Hp*-infected INS-GAS mice, a topic that warrants further investigation. Notably, a study examined the human gastric microbiota in 31 samples from different stages of the Correa cascade and found increased Shannon diversity during gastric cancer compared to gastritis or metaplasia (82); another study found increased phylogenetic diversity and richness (but not Shannon diversity) in the gastric microbiota from 12 human subjects with gastric cancer compared to 20 control subjects (83). Thus, the observation that bacteria other than *Hp* modulate disease pathogenesis in INS-GAS mice may be yet another way in which this model mimics human disease pathogenesis. However, the key takeaway from the INS-GAS model is that *Hp* infection is sufficient to accelerate disease progression compared to uninfected mice, decreasing the time of cancer onset from 20 months in uninfected mice to 7 months in conventionally housed mice and 11 months in gnotobiotic mice. Thus, this model again supports the overarching hypothesis that *Hp* is an active driver of gastric carcinogenesis, rather than a mere bystander.

### MNU chemical carcinogenesis model

Exposure to dietary nitrates is a potential risk factor for gastric cancer (84, 85) due to their conversion to carcinogenic N-nitroso compounds (86). Treatment of mice with the synthetic N-nitroso compound *N*-methyl-*N*-nitrosourea (MNU) mimics these exposures. Five weeks of MNU treatment in wild-type C57BL/6 mice caused a high incidence of gastric tumors (>80%), specifically in the antrum or lower part of the stomach. *Hf* infection increased the incidence to 100%, with a statistically significant increase in the number of tumors per animal (87). In addition, the combination of MNU treatment and *Hf* infection led to a synergistic decrease in the expression of *Tff1* (87), a peptide secreted by surface mucous cells that may act as a tumor suppressor and/or regulator of antrum stem cells (88). In another study, the same concentration of MNU caused gastric cancer in 27% of non-infected mice and 80% of *Hp*-infected mice, and a lower concentration of MNU did not cause gastric cancer in non-infected mice but caused gastric cancer in 43% of *Hp*-infected mice. Control mice that were infected with *Hp* but were not exposed to MNU did not develop metaplasia or cancer, demonstrating that cancer was dependent on interactions between *Hp* and MNU administration. As well, *Hp* impacted cancer morphology: all cancers caused by MNU alone were tubular adenocarcinomas, whereas in *Hp*-infected, MNU-treated mice, 20% of cancers were signet ring cell carcinomas, and the rest were tubular adenocarcinomas (89). *Hp* infection was also reported to exacerbate MNU-dependent gastric cancer development in gerbils (90).

### *Hp*+KRAS+ metaplasia model

KRAS is a small GTPase signaling protein of the Rat Sarcoma Virus (RAS) family that regulates cell survival, proliferation, and differentiation (91, 92). About 40% of gastric tumors have signatures of RAS activity, often due to amplifications of receptor tyrosine kinase (RTK)-RAS pathway genes (93, 94). In mice, induction of KRAS^G12D^ in the stomach using various Cre drivers rapidly induces clinically relevant metaplasia phenotypes (95–98). One such model is the *Mist1-Kras* mouse, in which subcutaneous tamoxifen induces KRAS^G12D^ in *Mist1*-expressing cells; in the stomach, these are isthmal progenitor cells and chief cells (95, 99), cell types that have each been argued to be the cell of origin of metaplasia (100–102). Within 1 month of KRAS^G12D^ induction, these mice develop a type of metaplasia called spasmolytic polypeptide (TFF2)-expressing metaplasia, or SPEM, which progresses to intestinal metaplasia and mild dysplasia over the next 2 to 3 months (95). SPEM is similar to the pseudopyloric metaplasia observed in the Correa cascade (10) and is a precursor lesion strongly associated with gastric cancer development (103).

**Fig 2:**
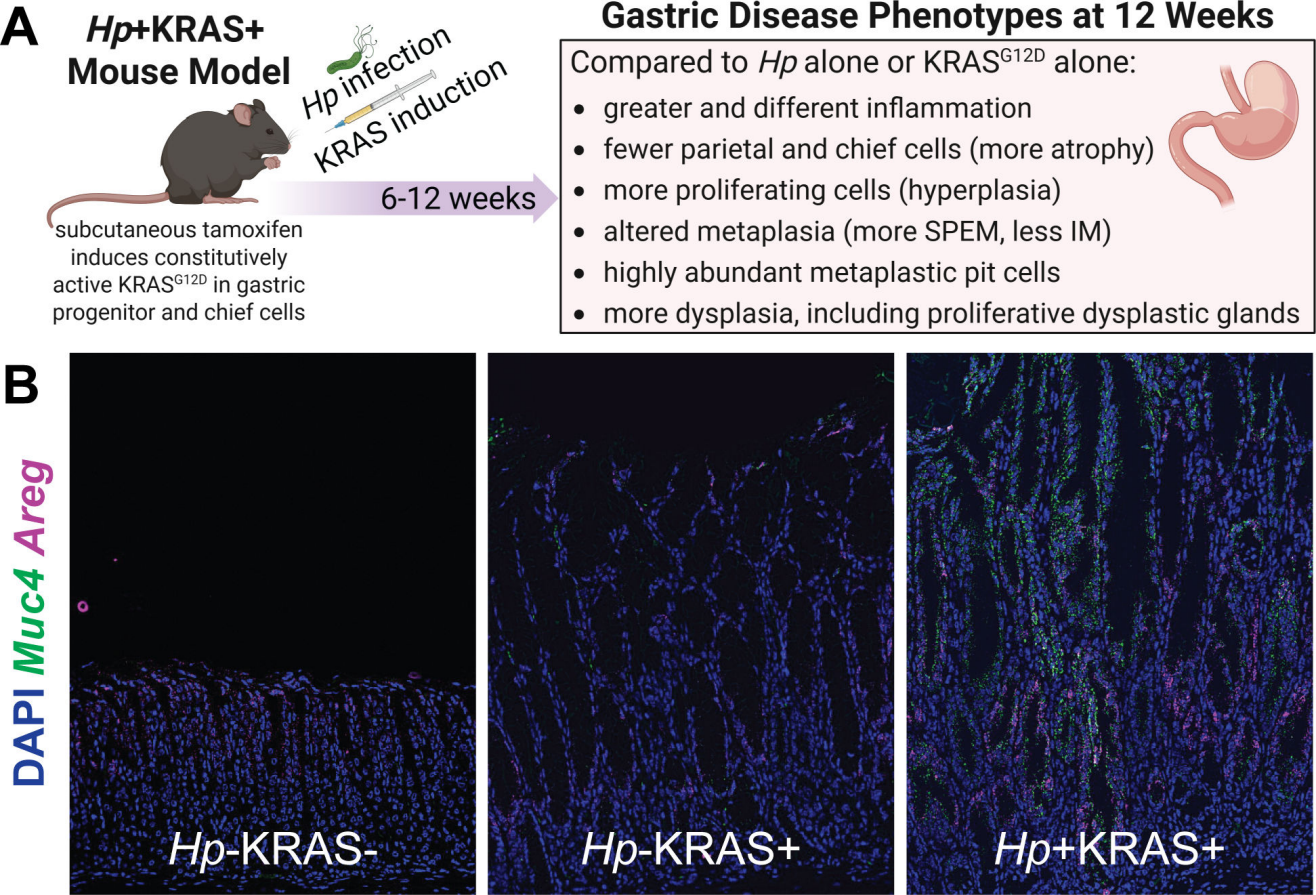
The *Hp*+KRAS+ mouse model shows that *Hp* infection significantly impacts metaplasia phenotypes. (**A**) In *Mist1-Kras* mice, subcutaneous tamoxifen is used to induce constitutively active KRAS^G12D^ in two cell types postulated to be the cell of origin of metaplasia, chief cells and isthmal progenitor cells. While induction of KRAS^G12D^ is sufficient to cause inflammation, metaplasia, and mild dysplasia within 12 weeks, we found that concomitant *Hp* infection alters and exacerbates these phenotypes. So-called “*Hp*+KRAS+ mice” had more severe hyperplasia and dysplasia and an altered trajectory of metaplasia (104), including the expansion of metaplastic pit cells (105). Also, acute *Hp* infections after KRAS^G12D^ induction provide a novel model system for evaluating bacterial determinants of colonization of the metaplastic stomach (109) and even for interrogating *Hp*’s interactions with other putative oncogenic microbes (110). (**B**) *In situ* hybridization was used to detect the metaplastic pit cell markers *Muc4* and *Areg* in gastric tissues from the indicated mouse groups after 12 weeks. Created in BioRender. O'Brien, V. (2026) https://BioRender.com/vtwvx7a.

We harnessed the *Mist1-Kras* model to test our overarching hypothesis that *Hp* can influence gastric preneoplastic progression (Fig. 2A). We evaluated stomachs 12 weeks after *Hp* infection or mock infection and KRAS^G12D^ induction or sham induction (104, 105). *Hp*+ mice had moderate inflammation, as expected given their gastric infection, but no metaplasia or dysplasia. KRAS+ mice had intestinal metaplasia, including mucus-filled cells with goblet morphology, in line with previous observations (95); these mice also had moderate inflammation, which is not unexpected given that KRAS^G12D^ induces inflammation (106). Critically, *Hp*+KRAS+ mice had far greater immunopathology, with sheets of immune cells in the lamina propria, loss of polarity of epithelial cells, and disrupted gland architecture with extensive branching and disorganized maturation. In a blinded analysis by a veterinary pathologist, *Hp*+KRAS+ mice had significantly increased inflammation, parietal cell loss, and hyperplasia compared to *Hp*+ or KRAS+ mice. Immunostaining for metaplasia markers suggested an altered trajectory of metaplasia, with KRAS+ mice skewing toward intestinal metaplasia and *Hp*+KRAS+ mice skewing toward SPEM (104). Intriguingly, several studies have reported that the association of SPEM with gastric cancer is equal to or even greater than that of intestinal metaplasia (103, 107, 108). Finally, *Hp*+KRAS+ mice had more severe dysplasia than KRAS+ mice and significantly more proliferating dysplastic glands. Thus, *Hp* infection plus KRAS^G12D^ expression accelerates gastric preneoplastic progression compared to KRAS^G12D^ alone and elicits greater inflammation than *Hp* infection or KRAS^G12D^ alone, in a manner that recapitulates human pathology. Gastric single-cell RNA-sequencing revealed that *Hp*+KRAS+ mice had a striking expansion of a previously undescribed cell type we termed “metaplastic pit cells” due to their genetic similarity to gastric pit (foveolar) cells coupled with their expression of metaplasia-associated genes such as the intestinal mucin *Muc4* and the growth factor amphiregulin, *Areg* (105) (Fig. 2B). In published human gastric scRNA-seq data sets, metaplastic pit cells were extremely rare in subjects with mild inflammation caused by *Hp* infection, and increased in abundance as disease severity increased. To expand on these computational analyses, we analyzed tissues from 47 subjects with gastric cancer. While MUC4 is not expressed in the healthy stomach, in 21 of 47 cancer samples, *MUC4*+ cells comprised 10% or more of the sample. In addition, *MUC4* expression was strongly correlated with cell proliferation and was associated with *Hp* infection status, though the latter association was not statistically significant, possibly due to the low prevalence of *Hp* in our cohort. Antibiotic therapy in *Hp*+KRAS+ mice both prevented and reversed these *Hp*-dependent phenotypes (104, 105), in line with the outcomes from antibiotic eradication studies described above. Thus, while studies in INS-GAS and MNU mice demonstrate that *Hp* infection accelerates gastric carcinogenesis, studies in *Mist1-Kras* mice show that *Hp* alters preneoplastic progression as well, skewing the balance of metaplastic lineages in the stomach (Fig. 2), with less SPEM, more intestinal metaplasia, and a substantial expansion of metaplastic pit cells.

*Hp* is a highly genetically diverse pathogen (111) that evolves within the human stomach over time to adapt to specific stomach niches (112–114). We used *Mist1-Kras* mice to mechanistically investigate host-pathogen interactions and within-host evolution in the context of the metaplastic stomach environment (109). We first induced KRAS^G12D^ to trigger metaplasia and then infected mice with *Hp* for 1 week. We used immunostaining to quantify bacteria within the gastric glands. In wild-type mice, at this acute time point, *Hp* was found predominantly in the glands of the antrum, the lower part of the stomach where glands do not produce gastric acid or digestive enzymes. However, in mice with SPEM or intestinal metaplasia, *Hp* very robustly colonized the corpus glands, the oxyntic glands of the stomach body that produce gastric acid and digestive enzymes. These studies used *Hp* strain PMSS1, which has an active *cag* type IV secretion system and causes inflammation in mice (65). To explore bacterial factors that could promote metaplastic gland colonization, we evaluated other *Hp* strains, including from the so-called “J99 collection” (113). *Hp* strains J99, SC4, and 10 others were isolated from an antrum biopsy collected in 1994 from a patient with a duodenal ulcer who then refused antibiotic treatment. By 2000, the patient had developed corpus-predominant gastritis and gastric atrophy, indicating worse disease. Multiple biopsies were collected at this time point, yielding 27 additional *Hp* isolates. Genetic analysis revealed that the year 2000 strains clustered into two subgroups with distinct phenotypes, including differences in the cell morphology and ability to induce inflammatory cytokines. Thus, this critical study demonstrates that *Hp* undergoes genetic adaptation during disease progression (113). We found that J99 and SC4 were relatively poor colonizers of mice, regardless of metaplasia status. Strains D1 and C2 are representative of the two subgroups of year 2000 strains, and we found that in mice, the D1 strain could colonize the healthy stomach (median titer 8 × 10^4^ colony-forming units [CFU]) and colonized the metaplastic stomach significantly better (median titer 1 × 10^6^ CFU). Thus, at least some *Hp* strains may flourish in the metaplastic stomach environment. The C2 strain had very heterogeneous colonization; about half of mice had no detectable CFU, but one mouse with metaplasia had a titer of 3 × 10^6^ CFU. We isolated an output strain from this mouse, which we termed “C2-adapted.” Intriguingly, this strain was much better suited to colonizing the mouse stomach. The median CFU of the C2-adapted strain was 5 × 10^4^ in healthy mice and 9 × 10^5^ in mice with metaplasia. Thus, we concluded that strain C2 was poised to adapt to *in vivo* colonization and that the adaptation favored colonization of the metaplastic stomach environment. Whole-genome sequencing revealed that the “stock” (parent) C2 strain had two alleles of the putative adhesin SabB; the predominant allele (variant 1) was present at a frequency of ~80% and variant 2 at a frequency of ~20%. In the C2-adapted strain, the ratio shifted, with variant 2 present at a frequency of ~40%–50%. Variant 2 likely arose from a recombination event with the adhesin SabA. *Ex vivo* binding assays showed that Δ*sabB* mutant strains adhered significantly less well to gastric tissue than wild-type strains did, supporting the hypothesis that SabB is an adhesin. In competitive infections, a Δ*sabB* mutant strain was attenuated in the healthy mouse stomach and very strongly attenuated in the metaplastic stomach environment. Together, these results demonstrate that *Hp* can quickly adapt to the metaplastic gastric environment via genetic modification (109). In line with our findings, another study used drug treatment to ablate parietal cells and induce SPEM in mice. *Hp* showed a tropism for metaplastic glands: in an *ex vivo* tissue adherence assay, *Hp* bound to the tops of the gastric glands in control-treated mice or mice that had recovered from SPEM, but it bound deeper in the glands, where SPEM cells are located, in drug-treated mice (115). The tropism was attributed to the high expression of sialyl-Lewis^X^, a receptor for the *Hp* adhesin SabA (116), in SPEM glands, and was supported by *in vivo* infection studies in mice. Taken together, these studies again challenge the notion that gastric cancer is an unintended consequence of *Hp* infection. Given that at least some *Hp* strains can robustly colonize metaplastic glands in mice and that *Hp* has at least one mechanism—adhesin gene conversion events—to favor this colonization, it seems likely that *Hp* has evolved to actively cause metaplasia.

## PERSPECTIVE

An analysis of global cancer data (“GLOBOCAN”) from 2018 estimated that 13% of all new cancers that year were attributable to infection (3). Of these 2.2 million new infection-associated cancers, the largest share (810,000, or 37%) were gastric cancers attributed to *Hp* infection. Thus, a better understanding of how and why *Hp* causes cancer is urgently needed. In Dr. Correa’s own words: “The [precancerous cascade] is initiated and sustained by infection with *Hp*” (117) (emphasis added). Therefore, research must go beyond investigating how *Hp* establishes gastric colonization and initiates inflammation; we must interrogate the complex host-pathogen interactions that occur in the later stages of disease in order to fully dissect the complexity of gastric cancer development in humans. Critically, rodent models have been invaluable for studying the bacterial and host determinants of infection—in the healthy stomach. In light of the fact that *Hp* can and does persist throughout the entire Correa cascade in at least some individuals and actively drives disease progression as evidenced by antibiotic eradication trials, we should consider the possibility that host-pathogen interactions differ in the metaplastic and/or cancerous stomach environment compared to the healthy stomach. Indeed, recent work shows that *Hp* has a tropism for metaplastic (SPEM) glands in mice (115), that some *Hp* strains can colonize the metaplastic mouse stomach better than the healthy mouse stomach (109), and that *Hp* can genetically adapt to preneoplastic changes, both in mice (109) and in humans (113). While numerous transgenic mouse models have given critical insights into gastric carcinogenesis, most have not evaluated how concomitant *Hp* infection impacts disease progression (118). Also, these mouse models are an excellent means for characterizing the interactions between *Hp* and other microbes that may modulate disease progression (79–81, 110). A greater understanding of host-pathogen interactions during late-stage disease may reveal new targets for anti-virulence therapies or even for a possible gastric cancer vaccine.

*Hp* was the only bacterial pathogen included in the GLOBOCAN analysis (3), and a critical limitation is the exclusion of certain cancers that we now appreciate can be caused by bacterial infections. Most notably, the analysis did not consider colorectal cancer to be infection-associated. However, at least three bacteria are linked with colorectal carcinogenesis: *pks*+ (colibactin-producing) *Escherichia coli*, enterotoxigenic *Bacteroides fragilis*, and *Fusobacterium nucleatum* (119, 120). Unlike gastric cancer, where 80% of cases are attributable to *Hp* infection (3), the fraction of colorectal cancers attributable to these bacteria is difficult to estimate. However, given the very high global incidence of colorectal cancer (more than 1.9 million new cases in 2022 [1]), coupled with the high incidence of gastric cancer, we can conclude that bacterial-associated cancers are a tremendous cause of global morbidity and mortality. Studies investigating bacterial origins of colorectal cancer should take into account the possibility that these bacteria may also be active drivers of carcinogenesis, like *Hp* is in at least some individuals.
